# Metallochaperones Are Needed for Mycobacterium tuberculosis and Escherichia coli Nicotinamidase-Pyrazinamidase Activity

**DOI:** 10.1128/JB.00331-19

**Published:** 2020-01-02

**Authors:** Patricia Sheen, Anuntxi Monsalve, Jhanina Campos, Rodolfo Huerta, Ricardo Antiparra, Héctor Arteaga, Patricia Duran, Carlos Bueno, Daniela E. Kirwan, Robert H. Gilman, Mirko Zimic

**Affiliations:** a Laboratory of Bioinformatics and Molecular Biology, Department of Cellular and Molecular Sciences, School of Science, Universidad Peruana Cayetano Heredia, Lima, Peru; b Infection and Immunity Research Institute, St. George’s, University of London, London, United Kingdom; c Department of International Health, School of Public Health, Johns Hopkins University, Baltimore, Maryland, USA; University of Illinois at Chicago

**Keywords:** *Mycobacterium tuberculosis*, PZA resistance, pyrazinamide, pyrazinoic acid, metallochaperone, metalloenzyme, resistance, mechanism of action, mechanism of resistance, ZnuA, Rv2059, reactivation, metal depletion

## Abstract

Mycobacterium tuberculosis nicotinamidase-pyrazinamidase (PZAse) is a metalloenzyme that catalyzes conversion of nicotinamide-pyrazinamide to nicotinic acid-pyrazinoic acid. This study investigated whether a metallochaperone is required for optimal PZAse activity. M. tuberculosis and Escherichia coli PZAses (PZAse-MT and PZAse-EC, respectively) were inactivated by metal depletion (giving PZAse-MT–Apo and PZAse-EC–Apo). Reactivation with the E. coli metallochaperone ZnuA or Rv2059 (the M. tuberculosis analog) was measured. This was repeated following proteolytic and thermal treatment of ZnuA and Rv2059. The CDC1551 M. tuberculosis reference strain had the Rv2059 coding gene knocked out, and PZA susceptibility and the pyrazinoic acid (POA) efflux rate were measured. ZnuA (200 μM) achieved 65% PZAse-EC–Apo reactivation. Rv2059 (1 μM) and ZnuA (1 μM) achieved 69% and 34.3% PZAse-MT–Apo reactivation, respectively. Proteolytic treatment of ZnuA and Rv2059 and application of three (but not one) thermal shocks to ZnuA significantly reduced the capacity to reactivate PZAse-MT–Apo. An M. tuberculosis Rv2059 knockout strain was Wayne positive and susceptible to PZA and did not have a significantly different POA efflux rate than the reference strain, although a trend toward a lower efflux rate was observed after knockout. The metallochaperone Rv2059 restored the activity of metal-depleted PZAse *in vitro*. Although Rv2059 is important *in vitro*, it seems to have a smaller effect on PZA susceptibility *in vivo.* It may be important to mechanisms of action and resistance to pyrazinamide in M. tuberculosis. Further studies are needed for confirmation.

**IMPORTANCE** Tuberculosis is an infectious disease caused by the bacterium Mycobacterium tuberculosis and remains one of the major causes of disease and death worldwide. Pyrazinamide is a key drug used in the treatment of tuberculosis, yet its mechanism of action is not fully understood, and testing strains of M. tuberculosis for pyrazinamide resistance is not easy with the tools that are presently available. The significance of the present research is that a metallochaperone-like protein may be crucial to pyrazinamide’s mechanisms of action and of resistance. This may support the development of improved tools to detect pyrazinamide resistance, which would have significant implications for the clinical management of patients with tuberculosis: drug regimens that are appropriately tailored to the resistance profile of a patient’s individual strain lead to better clinical outcomes, reduced onward transmission of infection, and reduction of the development of resistant strains that are more challenging and expensive to treat.

## Abstract (precis)

Tuberculosis is an infectious disease caused by the bacterium Mycobacterium tuberculosis and remains one of the major causes of disease and death worldwide. Pyrazinamide is a key drug used in the treatment of tuberculosis, yet its mechanism of action is not fully understood, and testing strains of M. tuberculosis for pyrazinamide resistance is not easy with the tools that are presently available. The significance of the present research is that a metallochaperone-like protein may be crucial to pyrazinamide’s mechanisms of action and of resistance. This may support the development of improved tools to detect pyrazinamide resistance, which would have significant implications for the clinical management of patients with tuberculosis: drug regimens that are appropriately tailored to the resistance profile of a patient’s individual strain lead to better clinical outcomes, reduced onward transmission of infection, and reduction of the development of resistant strains that are more challenging and expensive to treat.

## INTRODUCTION

Tuberculosis (TB) is a disease affecting 10.0 million people globally each year (1) and remains one of the major causes of disease and death worldwide. It is exacerbated by HIV-Mycobacterium tuberculosis coinfection (2) and by the emergence of multidrug-resistant (MDR) and extensively drug-resistant (XDR) M. tuberculosis strains in both industrialized and developing countries (2, 3).

Pyrazinamide (PZA) is a key drug used in the treatment of tuberculosis. Historically, its inclusion in first-line regimens enabled the duration of treatment to be shortened and led to a reduction in relapse rates (4, 5). It is active against slowly dividing M. tuberculosis bacteria and thus may be the most important drug in current and future TB treatment regimens (6, 7). The emergence of strains resistant to PZA represents an important public health problem, as PZA is a component of both first- and second-line treatment regimens. The number of patients with MDR TB, defined as the presence of resistance to both isoniazid and rifampin, is increasing globally (1), and additional resistance to PZA among MDR-TB patients was estimated to have occurred in 480,000 patients with TB in 2015 (8). A recent Tanzanian study found that 15 of 30 (50%) patients with MDR TB and 13 of 61 (21.3%) patients with drug-sensitive TB also had PZA resistance (9). In 2015, the rates of PZA resistance among new cases of MDR TB in Peru increased by 4%, and the percentage of MDR-TB cases with concomitant PZA resistance was almost 60% (10).

The mechanisms of action and of resistance to PZA in M. tuberculosis are incompletely understood. PZA is a prodrug that enters the mycobacteria by passive diffusion and is transformed in the cytoplasm into pyrazinoic acid (POA) by a nicotinamidase that also has nicotinamidase-pyrazinamidase (PZAse) activity (11). POA, the active drug, is expelled from the bacilli by an efflux system yet to be identified. In the acidic environment outside the bacilli, POA is protonated and then reenters the mycobacteria. Once back inside the bacilli, the protons are released, acidifying the cytoplasm and allowing POA to accumulate. This causes disruption in the mycobacterial membrane permeability and transport, leading to cell death (12, 13).

PZAse/nicotinamidase is a ubiquitous metalloenzyme present in prokaryotes and eukaryotes and expressed constitutively in M. tuberculosis (13, 14), Escherichia coli (15–17), Salmonella enteritidis serovar Typhimurium (17), Torula cremoris (15), and Borrelia burgdorferi (18). The physiological role of nicotinamidase is to convert nicotinamide (NAD) to nicotinic acid mononucleotide. Adenylation of this mononucleotide followed by amide formation completes the biosynthesis of NAD. NAD and NAD phosphate (NADP) are essential compounds in over 300 biochemical redox reactions (17).

It had previously been proposed that POA binds to the ribosomal protein RpsA and that this inhibits *trans* translation, which is lethal to the mycobacteria (19). According to this theory, PZA resistance may occur due to mutations in the RpsA C terminus that prevent the binding of POA (19), and in keeping with this, Shi et al. recently identified two mutations in the *rpsA* gene that were associated with PZA resistance (19). However, other data are contradictory: a study evaluating the interaction between RpsA and POA using isothermal titration calorimetry (ITC) found that deprotonation of POA in phosphate buffer was independent of RpsA (20).

Currently, the major mechanism of PZA resistance is thought to be loss of PZAse activity and therefore failure to hydrolyze PZA into POA. Defective PZAse is frequently found in PZA-resistant M. tuberculosis strains (14, 21–24), and several studies have demonstrated an association between PZA resistance and mutations in the *pncA* gene, which encodes PZAse (13, 14, 21–25). The same mechanism is responsible for the natural PZA resistance of Mycobacterium bovis and Mycobacterium kansasii (26, 27). This also suggests that *pncA* DNA sequencing could be used to predict resistance, provided that it is possible to predict the effect of mutations on PZAse function. A recent study that analyzed 10,209 M. tuberculosis isolates found that sequencing correctly predicted PZA resistance with 91.3% sensitivity and susceptibility with 96.8% specificity (28), suggesting that additional factors besides *pncA* mutations are also present.

Although PZAse is constitutively expressed in M. tuberculosis, alterations in the level of *pncA* expression are known to affect PZA resistance, even among PZAse wild-type strains (29, 30). Such alterations could impair intracellular PZAse activity and, as a consequence, the POA efflux rate, resulting in PZA resistance. Mutations affecting *pncA* expression include those affecting the *pncA* promoter and silent mutations that switch codons, and they are associated with low levels of tRNA.

Important molecular characteristics of M. tuberculosis PZAse (PZAse-MT) have been elucidated from crystallized homologous hydrolases, such as *N*-carbamoylsarcosine amidohydrolase (CHSase) from *Arthrobacter* (26% identical) (31), PZAse from Pyrococcus horikoshii (37% identical) (32), and PZAse from Acinetobacter baumannii (37% identical) (33), as well as from the recently crystallized structure of M. tuberculosis pyrazinamidase (34) and a theoretical analysis of a modeled structure (35). According to these studies, the catalytic cavity comprises an active site (D8, A134, and C138) and a metal-binding site (D49, H51, and H71). Du et al. (32) showed that Zn^2+^ was held in P. horikoshii PZAse crystals. Similarly, a PZAse from A. baumannii cocrystallized with nicotinamide contained Zn^2+^ and Fe^2+^ in a 1:1 ratio (33), while the M. tuberculosis PZAse was successfully crystallized with Fe^2+^ (34).

Metal ions are necessary for PZAse activity and have significant implications for PZA’s antibiotic mechanism of action (29, 32, 36–39). M. tuberculosis PZAse is inactivated by the removal of divalent ions and can be differentially reactivated (i.e., enzyme activity can be restored) (38, 40); Zhang et al. (38) observed reactivation by Fe^2+^ and Mn^2+^ when analyzed by inductively coupled plasma-optical emission spectroscopy (ICP-OES), and more recently, Sheen et al. (40) showed *in vitro* reactivation by Co^2+^, Mn^2+^, and Zn^2+^ but not Fe^2+^, Fe^3+^, or Mg^2+^. In this study, atomic absorption and X-ray fluorescence assays showed Zn^2+^ to be present in recombinant PZAse expressed in E. coli (PZAse-EC), and 300 times as much Zn^2+^ as enzyme was required for PZAse reactivation. Based on these observations, the authors proposed that *in vivo* PZAse obtains Zn^2+^ ions through an assisted process with the intervention of a metallochaperone. This is further supported by the observation that intracellular concentrations of proteins and metals exist at a ratio of 1:1 *in vivo* (41, 42).

A metallochaperone is a cellular transporter that delivers metal ions to proteins that require metals for their functional activity. The roles of metallochaperones and metal transporter proteins in M. tuberculosis, and specifically their relationships with antibiotic resistance, have not been studied in detail. However, metallochaperones have been described in other species (43), and their homologous proteins could similarly be explored in M. tuberculosis.

Metallochaperones of the TroA superfamily, such as ZnuA, are involved in Zn^2+^ importation by E. coli and other bacteria under low ion concentrations. This contributes to the growth and pathogenicity of the bacterium (44, 45). ZnuA is a key component of the ZnuABC zinc transporter, which has been well characterized (44–54). ZnuA captures free Zn^2+^ and delivers it to ZnuB, a transmembrane protein that carries Zn^2+^ into the cytoplasm; this is mediated by ZnuC, which supplies the necessary energy to complete the process (45).

In the recent annotation of the M. tuberculosis genome, Rv2059 was identified as a Zn^2+^ ABC-like transporter (55, 56) that may potentially facilitate interactions between M. tuberculosis PZAse and its Zn^2+^ metal cofactor.

In this study, we evaluated the hypothesis that ZnuA and its M. tuberculosis analog, Rv2059, are necessary for PZAse activity in E. coli and M. tuberculosis, respectively. To test this hypothesis, we measured the capacities of ZnuA and Rv2059 to facilitate the reactivation of metal-depleted E. coli and M. tuberculosis PZAses, respectively.

## RESULTS

### Measurement of Zn^2+^ content in ZnuA, ZnuA-Apo, and ZnuA-Tx by atomic absorbance.

The Zn^2+^ content was found to be 10-fold lower in metal-depleted ZnuA (ZnuA-Apo) than in either untreated ZnuA or heat-treated, EDTA-free ZnuA (ZnuA-Tx) (0.98 μM versus 11.11 μM and 9.52 μM, respectively) (Table 1).

**TABLE 1:** Concentrations of Zn^2+^ present in 10 μM (each) of the three protein variants ZnuA, ZnuA-Apo, and ZnuA-Tx

Metallochaperone state (10 μM)	[Zn^2+^] (μM)	[Zn^2+^]/[protein] ratio^*a*^
ZnuA	11.11	1:1
ZnuA-Apo	0.98	1:10
ZnuA-Tx	9.52	1:1

a Ratio of moles of Zn^2+^ to moles of protein.

### Kinetic parameters of PZAse-EC.

According to the linear regression model, for each unit that the inverse of the substrate concentration increased, the inverse of the velocities increased by 0.41 ± 0.05 μmol min^−1^ mg^−1^. The kinetic parameters of the PZAse-EC were as follows: Michaelis constant (*K_m_*), 0.36 ± 0.12 mM; catalytic constant (*K*_cat_), 873.16 ± 111.93 min^−1^; enzymatic activity, 42.74 ± 8.12 μM POA mg PZAse^−1^ min^−1^; *V*_max_, 0.65 ± 0.08 μmol min^−1^ mg^−1^; and enzymatic efficiency, 2,442.96 ± 588.58 mM^−1^ min^−1^, with a 95% confidence interval.

### Reactivation of PZAse-EC–Apo with Zn^2+^ and ZnuA.

**FIG 1:**
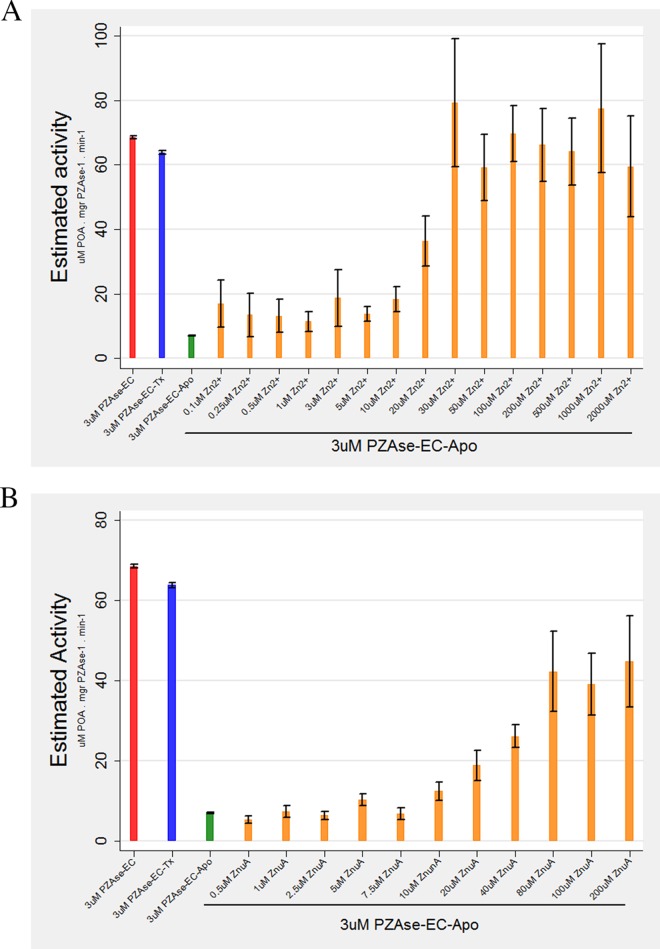
(A) Reactivation of PZAse-EC–Apo with Zn^2+^. (B) Reactivation of PZAse-EC–Apo with ZnuA. The bars represent mean estimated activity, and the whiskers represent 95% confidence intervals.

The enzyme activity of metal-depleted inactivated E. coli PZAse (PZAse-EC–Apo) was increased by the addition of both Zn^2+^ and ZnuA. PZAse-EC–Apo treated with Zn^2+^ reached 100% reactivation (i.e., reached 100% of the enzymatic activity of untreated PZAse-EC) with 30 μM Zn^2+^ (79 μM POA mg PZAse^−1^ min^−1^) (Fig. 1A). This showed a positive tendency when the Zn^2+^ concentration was increased, as determined by linear regression (*P* = 0.0001).

PZAse-EC–Apo treated with ZnuA (200 μM) was reactivated to a maximum of 65% (44.7 μM POA mg PZAse^−1^ min^−1^) (Fig. 1B). Restoration of enzyme activity occurred with 40 μM, 80 μM, 100 μM, and 200 μM ZnuA, with corresponding activities of 26.1, 42.2, 39.1, and 44.7 μM POA mg PZAse^−1^ min^−1^. ZnuA concentrations below 10 μM did not reactivate PZAse-EC–Apo. ZnuA showed a positive tendency in the reactivation of PZAse-EC–Apo, as determined using linear regression (*P* < 0.0001).

### Reactivation of PZAse-MT–Apo with Zn^2+^ and Rv2059.

**FIG 2:**
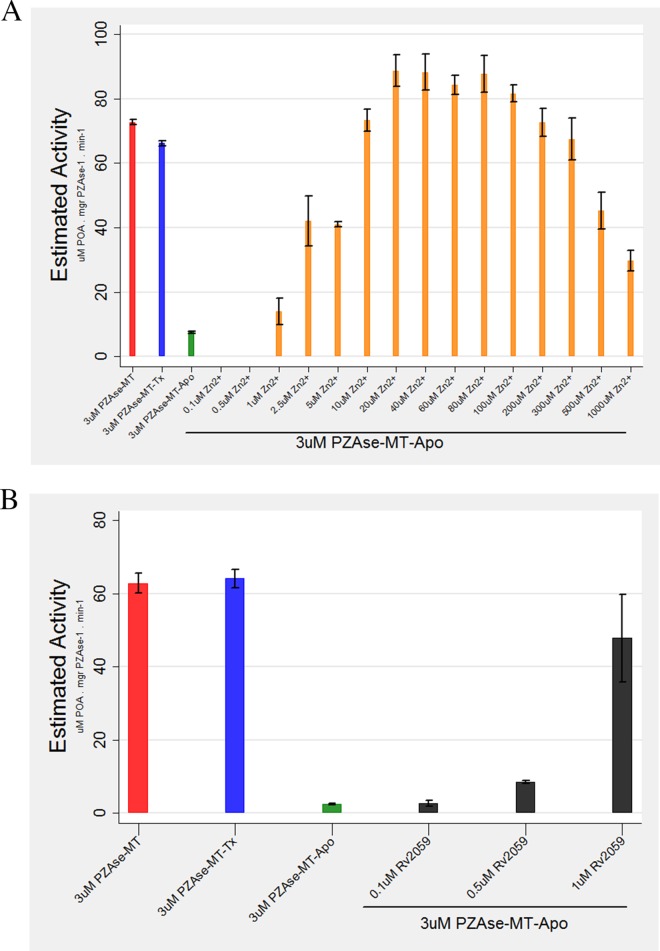
(A) Reactivation of PZAse-MT–Apo with Zn^2+^. (B) Reactivation of PZAse-MT–Apo with Rv2059. The bars represent mean estimated activity, and the whiskers represent 95% confidence intervals.

Metal-depleted inactivated M. tuberculosis PZAse (PZAse-MT–Apo) treated with Zn^2+^ reached 100% reactivation with 10 μM Zn^2+^ (73.39 μM POA mg PZAse^−1^ min^−1^) (Fig. 2A). PZAse-MT–Apo reactivation showed a positive tendency with increasing Zn^2+^ concentrations up to 100 μM and a negative tendency thereafter, as determined using linear regression (*P* = 0.0001).

Concentrations of Rv2059 below 0.5 μM failed to achieve reactivation of PZAse-MT–Apo. At 0.5 μM Rv2059, 16% of enzyme activity was recovered (8.8 μM POA mg PZAse^−1^ min^−1^), and with 1 μM Rv2059, this reached 69% (47.8 μM POA mg PZAse^−1^ min^−1^) (Fig. 2B).

### Reactivation of PZAse-MT–Apo with ZnuA and ZnuA-Apo.

**FIG 3:**
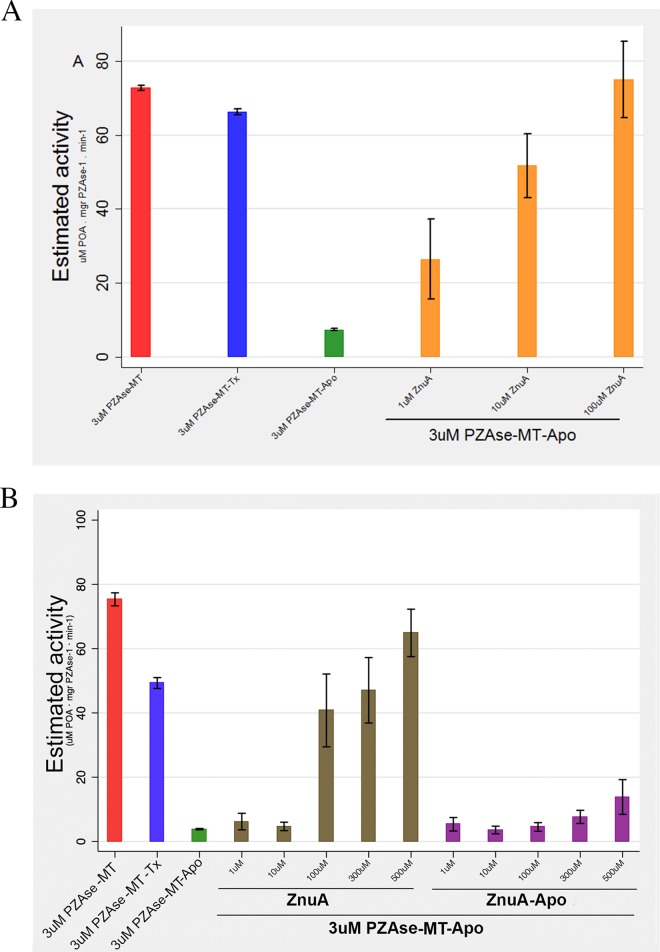
(A) Reactivation of PZAse-MT–Apo with ZnuA. (B) Reactivation of PZAse-MT–Apo with ZnuA and ZnuA-Apo. The bars represent mean estimated activity, and the whiskers represent 95% confidence intervals.

PZAse-MT–Apo activity (7.4 μM POA mg PZAse^−1^ min^−1^) increased with the addition of 1 μM ZnuA to 26.5 μM POA mg PZAse^−1^ min^−1^, which is equivalent to 55.4% of the activity achieved by Rv2059 (47.8 μM POA mg PZAse^−1^ min^−1^); 10 and 100 μM ZnuA resulted in activities of 51.7 and 75 μM POA mg PZAse^−1^ min^−1^, respectively (Fig. 3A).

To test the resilience of the metallochaperone, the assay was repeated with 1 mM phosphate buffer. PZAse-MT–Apo activity (7.4 μM POA mg PZAse^−1^ min^−1^) increased with the addition of 100, 300, and 500 μM ZnuA, resulting in activities of 41, 47, and 64 μM POA mg PZAse^−1^ min^−1^, respectively (Fig. 3B). The activity of PZAse-MT–Apo was not affected by the addition of 1 or 10 μM ZnuA when 1 mM phosphate buffer was used, in contrast to reactivation under conditions of 50 mM phosphate buffer, which showed increased reactivation with all ZnuA concentrations used. This suggests that a structural factor is involved in the reactivation of PZAse-MT–Apo by ZnuA.

Under weak buffer conditions, ZnuA reactivated PZAse-MT–Apo to a greater extent than ZnuA-Apo (*P* < 0.0001): 100 μM, 300 μM, and 500 μM ZnuA reactivated PZAse-MT–Apo by 82%, 95%, and 135% more than ZnuA-Apo (Fig. 3B).

### Effects of proteolytically inactivated ZnuA and Rv2059 on the reactivation of metal-depleted PZAse-MT–Apo.

Degradation of ZnuA and Rv2059 following proteolytic treatment was verified by SDS-PAGE followed by staining with Coomassie brilliant blue for ZnuA and silver nitrate for Rv2059.

**FIG 4:**
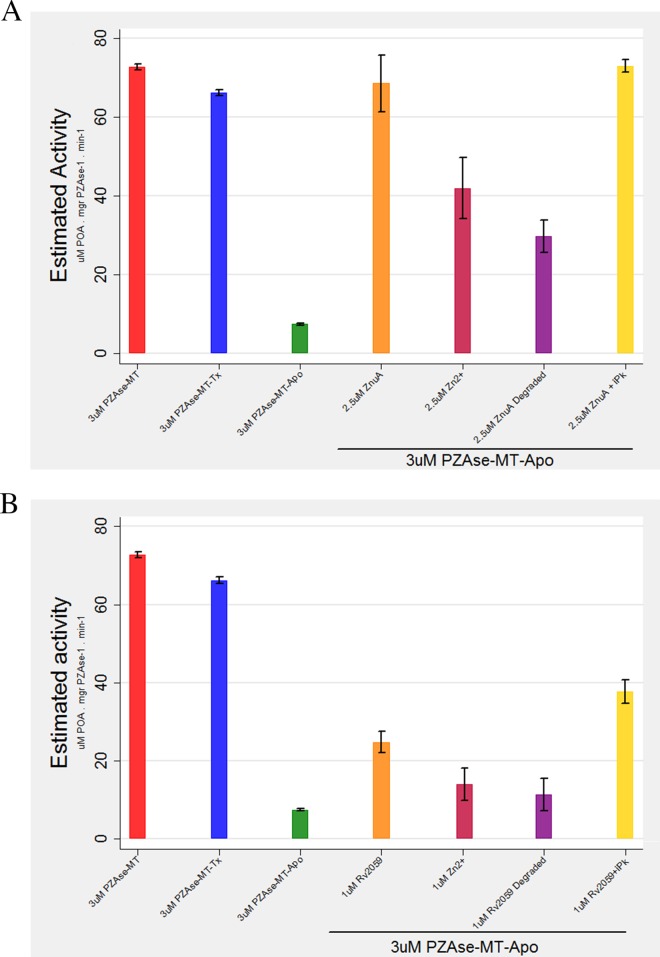
(A) Reactivation of PZAse-MT–Apo with ZnuA versus degraded ZnuA. (B) Reactivation of PZAse-MT–Apo with Rv2059 versus degraded Rv2059. The colors represent different experimental conditions: red, PZAse-MT; blue, PZAse-MT–Tx; green, PZAse-MT–Apo; orange, ZnuA or Rv2059; magenta, Zn; purple, proteolytically degraded ZnuA or Rv2059; and yellow, ZnuA or Rv2059 and thermally inactivated proteinase K (iPk). The bars represent mean estimated activity, and the whiskers represent 95% confidence intervals.

**(i) Comparison of the effects of proteolytically degraded ZnuA versus ZnuA on recovered activity of PZAse-MT–Apo.** ZnuA (2.5 μM) led to an increase in PZAse-MT–Apo activity by 66.6 μM POA mg PZAse^−1^ min^−1^, whereas degraded ZnuA (2.5 μM) increased activity by 30.9 μM POA mg PZAse^−1^ min^−1^. The activity recovered by degraded ZnuA was similar to that generated by 2.5 μM Zn^2+^ (1.9 μM POA mg PZAse^−1^ min^−1^). Thermally inactivated proteinase K and ZnuA (2.5 μM) increased PZAse-MT–Apo activity to 73 μM POA mg PZAse^−1^ min^−1^. The addition of inactive proteinase K did not reduce reactivation (Fig. 4A).

**(ii) Comparison of the effects of proteolytically degraded Rv2059 versus Rv2059 on the capacity to recover activity of PZAse-MT–Apo.** Rv2059 (1 μM) reactivated PZAse-MT–Apo activity to 24.8 μM POA mg PZAse^−1^ min^−1^ and degraded Rv2059 (1 μM) to 12 μM POA mg PZAse^−1^ min^−1^. The activity generated by 2.5 μM Zn^2+^ was 16 μM POA mg PZAse^−1^ min^−1^. Thermally inactivated proteinase K with Rv2059 reactivated PZAse-MT–Apo to 37.6 μM POA mg PZAse^−1^ min^−1^. As observed above, proteinase K did not reduce reactivation; on the contrary, trace concentrations of ions conferred a boost to PZAse-MT–Apo activity (Fig. 4B).

### Effect of thermal inactivation of ZnuA on reactivation of metal-depleted PZAse-MT–Apo.

PZAse-MT–Apo enzymatic activity was partially recovered by thermally inactivated ZnuA: the reactivation of PZAse-MT–Apo by ZnuA treated with one thermal shock was 30% less than when ZnuA without any treatment was used at the same concentration, whereas reactivation by ZnuA that had been inactivated with three thermal shocks was 43%. The Tris-HCl buffer-only control did not reactivate the enzyme.

### Structural model of ZnuA/Rv2059 and docking with PZAse-MT.

**FIG 5:**
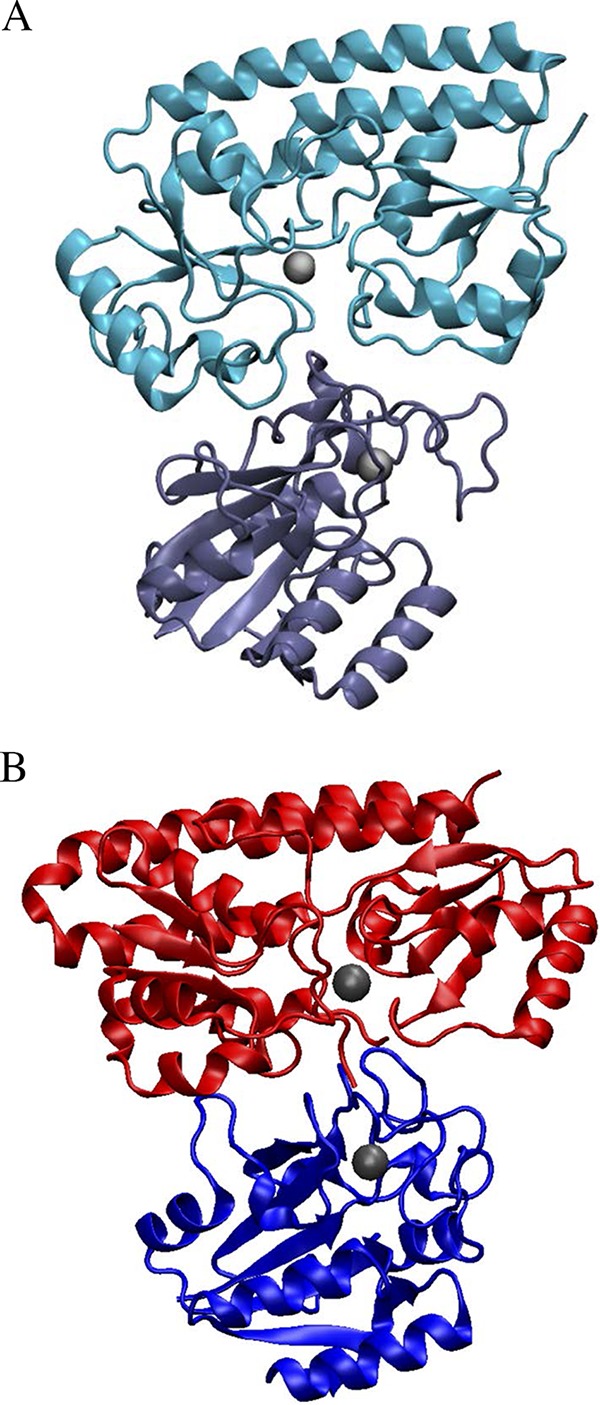
(A) Possible binding between Rv2059 (top) and PZAse (bottom). The distance between the two metallic clusters, shown as gray spheres, is 21 nm. (B) Possible binding between E. coli ZnuA (2OSV), shown in red, and PZAse (3PL1), shown in blue. The distance between the metallic centers, shown as gray spheres, is 20 nm.

The predicted interactions between PZAse-MT and ZnuA or Rv2059 showed a favorable direct communication with no sterical impediments between the metal-binding sites of the two proteins. The distance between the metal-binding sites in the docking of ZnuA and Rv2059 with PZAse was around 20 nm. Representatives of the highest-scoring structures from both clusters are shown in Fig. 5.

### Wayne test, PZA susceptibility MIC, and POA efflux rate.

The POA efflux rates for the M. tuberculosis wild-type strain CDC1551 (0.023 μM POA/h) and the CDC1551-Rv2059-KO (knockout) strain (0.026 μM POA/h) were not significantly different (*P* = 0.27). No obvious difference was observed in the Wayne test or in MGIT (see below) PZA susceptibility testing between the two strains. This indicates that the CDC1551-Rv2059-KO strain is no more resistant to PZA than the wild type, and therefore, *in vivo* Rv2059 cannot be the only metallochaperone involved in the activation of PZAse.

## DISCUSSION

This study demonstrates for the first time that a metallochaperone-like protein significantly increased the enzymatic activity of M. tuberculosis PZAse *in vitro.* In our experiments, the metallochaperone Rv2059 effected the reactivation of metal-depleted PZAse from M. tuberculosis. It may act in a similar manner *in vivo*, regulating intracellular PZAse function, and may therefore play a crucial role in PZA’s mechanisms of action and of resistance. As Rv2059 was shown not to be essential to PZAse activity *in vivo*, it may be one of a number of metallochaperones with this function.

M. tuberculosis PZAse has nicotinamidase activity: it hydrolyzes nicotinamide to nicotinic acid and ammonia in the b-NAD (NAD^+^) salvage pathway (57). NAD modulates antioxidation and oxidative stress, and the NADH/NAD^+^ ratio is a measure of cellular reducing potential. Intracellular redox changes in M. tuberculosis have been previously reported (58); cultures of M. tuberculosis in the presence of certain antibiotics were associated with a NADH/NAD^+^ ratio change of 2.65-fold compared to untreated mycobacteria. Studies in mouse livers indicate that excessive intracellular NADH can produce “reductive stress,” which may result from its capacity to induce the release of ferrous iron from ferritin, an enzyme found in M. tuberculosis. Any evolutionary advantage or biological role of PZAse as a monomer or homodimer *in vivo* might thus be associated with its specific nicotinamidase activity (59). Therefore, it is likely that Rv2059 or other, similar metallochaperones may participate in activating the nicotinamidase activity *in vivo* and thus regulate the intracellular reductive/oxidative stress.

Pyrazinamidase is a nonessential enzyme in NAD synthesis, despite having a fundamental role in NAD recycling, because M. tuberculosis presents high plasticity for NAD synthesis. However, the NAD salvage pathway has been shown to be increased during infection (57). This could be due to RV2059 capturing zinc ions from the extracellular environment to activate PZAse.

As an active pyrazinamidase is fundamental to PZA susceptibility, mutated or ineffective RV2059 or other, similar metallochaperones could be markers of PZA resistance. On the other hand, supplementing PZA-resistant M. tuberculosis with RV2059 (or other, similar metallochaperones) or zinc could possibly induce susceptibility, assuming that the *pncA* gene has no mutations. Understanding this would have significant clinical consequences, as PZA is a key component of TB treatment regimens and adequate tools to test for resistance are currently lacking.

The E. coli homologue ZnuA was also capable of reactivating PZAse-EC–Apo, but the effect was far less potent than in M. tuberculosis: 10 to 20 times more ZnuA than PZAse-EC–Apo was required to achieve reactivation, while Rv2059 was found to reactivate PZAse-MT–Apo at a third of its concentration. This suggests that Rv2059 could have a “natural” metallochaperone function in the activation of PZAse in M. tuberculosis, whereas ZnuA would not be expected to share this function in E. coli, as such high concentrations are unlikely to occur *in vivo*.

Proteolytic-degradation assays confirmed the importance of the structure of the metallochaperone by showing that after proteolytic degradation, the capacity of RV2059 to activate metal-depleted PZAse was greatly reduced. In fact, the activation capacity of the proteolytically degraded Rv2059 was similar to that of the corresponding equimolar concentration of free Zn^2+^ that would have been released by the degradation of the protein. This effect was observed in both the E. coli and the M. tuberculosis metallochaperone/PZAse systems.

The fact that proteolytically degraded Rv2059 and ZnuA were able to reactivate PZAse-MT–Apo similarly to an equivalent concentration of zinc suggests that these metallochaperones coordinate zinc in equimolar concentrations. Confirming this finding, the atomic absorbance analysis of ZnuA showed a Zn^2+^/ZnuA ratio close to 1. In addition, the three-dimensional (3D) structure models of ZnuA and Rv2059, which comprise one His-His-Glu metal-binding site, as shown in other studies (50), also suggest a 1:1 Zn^2+^/ZnuA molar ratio. The measured Zn^2+^/ZnuA ratio that is close to 1 also suggests that it is unlikely that other metals could have been coordinated by these metallochaperones during standard protein overexpression in E. coli when producing the recombinant protein.

Based on the results obtained following metal depletion of ZnuA in the reactivation of PZAse-MT–Apo, we conclude that the presence of Zn^2+^ is necessary for ZnuA to optimally reactivate PZAse-MT–Apo. This allows us to presume the existence of a metallochaperone with a similar, as yet unreported role in the activity of PZAse in tuberculosis.

According to the theoretical annotation of Rv2059, together with ZnuA, it belongs to the group of proteins known as the TroA superfamily, which are transporters of Zn^2+^ and Mn^2+^ (55) and intervene in the importation of Zn^2+^ by the ABC system (60). In addition, ZnuA and Rv2059 are regulated by the Zur protein, which promotes the transcription of both coding genes at low concentrations of Zn^2+^ (49, 55, 61). The sequence alignment between Rv2059 and ZnuA showed a conserved domain between the histidines at positions 60 and 143 of ZnuA, which are involved in the coordination of Zn^2+^, and histidines at positions 137 and 197 of Rv2059.

Of note, ZnuA was highly thermoresistant. One thermal shock (100°C for 30 min) only minimally affected the metallochaperone’s activity, but three consecutive thermal shocks were able to reduce PZAse-MT–Apo reactivation to 43% compared to reactivation by untreated ZnuA (83%). This indicates that the function of ZnuA is not significantly altered by high temperatures, and it therefore possesses high structural stability, which can be affected only by proteolytic enzymes such as proteinase K (22% reactivation). Other studies have reported that the thermal stability of other metallochaperones is increased in the presence of divalent ions (62).

Phosphate buffer conditions influence the recovery of PZAse activity mediated by ZnuA. When reactivation of PZAse with ZnuA was evaluated under low-molarity buffer conditions (0.8 to 1 mM), a small amount of PZAse-MT activity was recovered (Fig. 4), but full PZAse-MT reactivation was achieved only with an optimum buffer concentration of 50 mM. Fifty millimolar buffer alone was not able to reactivate PZAse-MT–Apo, precluding the possibility that the buffer itself contains traces of metal ions that are able to reactivate PZAse.

It is known that variations in pH affect both the activity and the stability of an enzyme (63). We believe that the low-molarity conditions during the reactivation of PZAse-MT–Apo by ZnuA might not be enough to regulate the pH within a range for proper structure/function for either or both PZAse and ZnuA.

Our results show that low concentrations of Zn^2+^ (10 to 80 μM) are capable of optimally reactivating PZAse, which has never been shown before. It had previously been reported (38, 40) that 1.5 mM Zn^2+^ was the optimum concentration needed to regain PZAse activity. However, that study did not evaluate as many points in the range of 1 μM to 2,000 μM Zn^2+^ as are reported here; the points that coincide with those in this study are consistent. On the other hand, our results show that the reactivation of PZAse-MT–Apo with Zn^2+^ follows a nonlinear response and is reduced at Zn^2+^ concentrations greater than 100 μM.

Other studies have shown that metalloenzymes can be reactivated by metal cofactors under saturating conditions with concentrations close to equimolar (64, 65), and therefore, concentrations higher than 100 μM Zn^2+^ for 3 μM PZAse may cause oversaturation and loss of activity.

Our results provide evidence that the protein Rv2059 functions as a facilitator of Zn^2+^ for the activation of M. tuberculosis PZAse *in vitro*. Rv2059 may therefore be a divalent cation metallochaperone that could participate in the mechanism of action of PZA in M. tuberculosis but is not likely to participate in the mechanism of resistance. The evaluation of Rv2059 activity *in vivo* using a knockout strain showed no alteration in bacterial POA production. Thus, there may be multiple metallochaperones with effects on PZAse activity similar to or perhaps even more potent than the action of Rv2059. Other metallochaperones that play an important role in zinc homeostasis, particularly relating to the organism’s survival in the host, have been identified in other pathogenic bacteria (66, 67). Regarding the possibility that Rv2059 (or other, similar metallochaperones) is an alternative drug target that could have an effect similar to that of PZA, blocking Rv2059 with a specific drug would result in a less active PZAse and thus in PZA resistance. However, other, similar metallochaperones may be involved in the activation of essential metalloenzymes. In those cases, metallochaperones may constitute potentially important drug targets. Further studies are needed to confirm our findings and also to further our understanding of the importance of metallochaperones to mechanisms of mycobacterial resistance to PZA.

## MATERIALS AND METHODS

### Cloning of coding genes *pncA* from M. tuberculosis and E. coli, *znuA* from E. coli, and *Rv2059* from M. tuberculosis.

The M. tuberculosis and E. coli *pncA*, M. tuberculosis *Rv2059*, and E. coli *znuA* genes were amplified by PCR using oligonucleotides with restriction sites for NcoI and XhoI. For the M. tuberculosis H37Rv *pncA* gene, primer pair 5′ CCC CCA TGG GCC GGG CGT TGA TCA TC and 5′ CCC CTC GAG GGA GCT GCA AAC CAA CTC was used to clone a 575-bp fragment. For the E. coli K-12 *pncA* gene, primer pair 5′ AAA CAT ATG CCC CCT CGC GC and 5′ AAA CTC GAG CCC CTG TGT CTC TTC CC was used to clone a 642-bp fragment. For the E. coli W3110 *znuA* gene, primer pair 5′ AAA CAT ATG TTA CAT AAA AAA ACG CTT CTT TTC G and 5′ CCT CGA GAT CTC CTT TCA GGC AGC TC was used to clone a 933-bp fragment. For the M. tuberculosis H37Rv *Rv2059* gene, primer pair 5′ CAT GCC ATG GGC AAA TCC GCC ATC CAT C and 5′ CGG CTC GAG AAG TGG CCG AAA GCG AG was used to clone a 1,350-bp fragment. After amplification and double digestion of the purified genes, the gene fragments were inserted into the pET28a plasmid using T4 DNA ligase (NE BioLabs, Ipswich, MA) for 16 h at 16°C. For purification, six histidines were added to the carboxy-terminal end. E. coli NovaBlue cells (Novagen, San Diego, CA) were transformed directly from the ligation reaction using a heat-based protocol (40). Plasmid DNA was extracted using the QIAprep Spin Miniprep kit (Qiagen, Valencia, CA) and sequenced in an ABI Prism 3100 genetic analyzer (Applied Biosystem, Foster City, CA) in both directions to ensure that the gene was in frame.

### Production of the recombinant proteins PZAse-MT and PZAse-EC and ZnuA from E. coli.

The carboxy-terminal hexahistidine-tagged proteins PZAse-MT and PZAse-EC and ZnuA from E. coli were expressed in BL21(DE3)(pLysS) cells (Novagen), as described previously (40). Isopropyl-β-d-thiogalactoside was added to fresh overnight cultures at a final concentration of 1 mM and incubated for 4 h at 37°C. Expression was confirmed by SDS‐PAGE and Western blotting. The cells were harvested by centrifugation at 4,830 × *g* at 4°C for 10 min, followed by resuspension of the pellet in 20 ml binding buffer (20 mM imidazole, 0.5 M NaCl, 20 mM phosphate buffer, pH 7.4). The suspension was frozen at −70°C and thawed at 37°C three times and then sonicated using a S3000 sonicator (Misonix, Farmingdale, NY) at power level 3 for 3 cycles on ice. Each cycle was 1 min long, with alternating on/off periods of 1 s each. The supernatant was centrifuged at 17,572 × *g* at 4°C for 30 min and then loaded on a 5-ml HisTrap (histidine-tagged) protein purification column (Pharmacia, Piscataway, NJ) and washed with 40 mM imidazole, 0.5 M NaCl, and 20 mM phosphate buffer, pH 7.4. Aliquots of the fractions obtained were analyzed by SDS-PAGE and Western blotting. The primary antibody used for Western blotting was mouse anti‐His monoclonal antibody (MAb) (GenScript; A00186). Fractions containing pure proteins were combined. The purified protein was concentrated 10 times and washed 3 times with 20 mM Tris-HCl, pH 7.9, by filtration using cellulose membranes with a 10-kDa filtering limit in an Ultracel Amicon ultrafiltration system (Millipore, Billerica, MA) at 4°C. The protein concentration was determined by the Bradford method (68) or by bicinchoninic acid (BCA) protein assay using bovine serum albumin (BSA) as a standard.

Expression of Rv2059 in our laboratory was unsuccessful; therefore, it was purchased from GenScript (Piscataway, NJ). We received 1 ml of Rv2059 at a concentration of 0.6 mg/ml in PBS, pH 7.4. The purity was 95%, according to SDS-PAGE and Western blot assays.

### Kinetic parameters of PZAse-EC and PZAse-MT.

The kinetic parameters of PZAse-EC were calculated using a modified PZA hydrolysis reaction (32). PZA at a range of concentrations (0 μM to 10 μM) was incubated with 1 mM PZAse-EC in 50 mM sodium phosphate buffer, pH 6.5, for 1 min; 10 μl of 20% FeNH_4_(SO_4_)_2_ and 890 ml of 100 mM glycine-HCl buffer, pH 3.4, were added to stop the reaction, and the optical density was measured at 450 nm in a 96-well plate. We calculated the *K_m_* to measure the affinity of PZAse for PZA, the *K*_cat_, and the enzymatic efficiency (Eff) as *K*_cat_/*K_m_* (69, 70). The enzyme activity was estimated as the quantity of POA produced in a reaction of 1 min divided by the total quantity of enzyme. The data were adjusted by linear regression to the Lineweaver-Burk plot (1/*V* versus 1/[*S*], where *V* is the velocity of production of POA and [*S*] is the concentration of substrate PZA) and by nonlinear regression to the Michaelis-Menten equation (71). The kinetic parameters of PZAse-MT have been previously measured and reported by Sheen et al. (29).

### Inactivation of PZAse-MT and PZAse-EC by metal depletion (chelation).

Ions were removed from the two PZAses using the chelating agent EDTA. PZAse (0.5 ml; 700 μM) in phosphate buffer (100 mM; pH 6.4) and EDTA (80 mM; pH 8) were incubated overnight at room temperature. PZAse without EDTA was included as a chelation control. The EDTA was removed by 3 washes with 100 mM phosphate buffer, pH 6.4, using ultrafiltration until 1 ml of PZAse in the apoprotein state (PZAse-MT–Apo or PZAse-EC–Apo) and 1 ml of PZAse control (PZAse-MT–Tx or PZAse-EC–Tx) were obtained. Aliquots of 500 μl were stored at –20°C. The protein concentration was measured using the Bradford assay, and chelation was confirmed through a quantitative Wayne assay.

### Chelation of ZnuA.

ZnuA (2 ml; 400 μM) was incubated in Tris-HCl (30 mM; pH 7) and EDTA (80 mM; pH 8) overnight at 4°C. An EDTA-free control was also used. The samples were washed 3 times with Tris-HCl (30 mM; pH 7) by ultrafiltration until 1 ml of ZnuA in an apoprotein state (ZnuA-Apo) and 1 ml of ZnuA with incubation treatment only (ZnuA-Tx) were obtained. The concentrations of the proteins were measured using the Bradford assay, and 500-μl aliquots were stored at −20°C.

Metal depletion of ZnuA was confirmed by measuring the Zn^2+^ concentrations in 10 ml of 10 μM ZnuA, ZnuA-Apo, and ZnuA-Tx in 30 mM Tris-HCl buffer, pH 7, using atomic absorption spectroscopy (AAS). A standard curve was constructed using known quantities of ZnSO_4_·7H_2_O. A buffer-only control was included to rule out the presence of the ion in the buffer.

### Reactivation of metal-depleted PZAse-EC–Apo with Zn^2+^ and ZnuA.

PZAse-EC–Apo (3 μM) was incubated with concentrations of Zn^2+^ (ZnSO_4_·7H_2_O) ranging from 0.1 μM to 2,000 μM or with ZnuA metallochaperone at concentrations ranging from 0.5 μM to 200 μM for 30 min, with 50 mM phosphate buffer. PZA (20 mM) was added, and after 3 min, the reaction was terminated with glycine-HCl, pH 3.4. POA produced after the hydrolysis of PZA was revealed with ammonium ferrous sulfate, as described by Sheen et al. (40). The absorbance of this modified Wayne assay (72) was read at 450 nm using a spectrophotometer. Enzyme activity was then estimated as described above.

### Reactivation of metal-depleted PZAse-MT–Apo with Zn^2+^ and Rv2059.

PZAse-MT–Apo (3 μM) was reactivated with increasing concentrations of Zn^2+^ (ZnSO_4_·7H_2_O) ranging from 0.1 μM to 1,000 μM Zn^2+^ or with Rv2059 metallochaperone (0.1 μM, 0.5 μM, and 1 μM) for 30 min, with 50 mM phosphate buffer; 20 mM PZA was then added for 3 min. The reaction was stopped with glycine-HCl, pH 3.4, and revealed with ammonium ferrous sulfate, as described above for PZAse-EC–Apo.

### Reactivation of metal-depleted PZAse-MT–Apo using ZnuA and ZnuA-Apo.

In order to evaluate whether the E. coli metallochaperone ZnuA was also able to reactivate PZAse from M. tuberculosis, 3 μM PZAse-MT–Apo was titrated with ZnuA at concentrations of 200 μM, 100 μM, 10 μM, and 1 μM using the method described above. In addition, to verify the importance of Zn^2+^ to the reactivation capacity of ZnuA, a reactivation assay was performed using ZnuA-Apo.

Next, we investigated whether the functional stability of ZnuA, previously tested in 50 mM phosphate buffer at pH 6.4, was maintained under low-molarity buffer (1 mM). PZAse-MT–Apo (3 μM) was titrated in 0.8 to 1 mM phosphate buffer, pH 6.4, with ZnuA or ZnuA-Apo at concentrations of 500 μM, 300 μM, 100 μM, 10 μM, and 1 μM. The activity for each of the reactions was measured quantitatively after incubation for 30 min at 37°C.

### Effects of proteolytically inactivated ZnuA and Rv2059 on reactivation of metal-depleted PZAse-MT–Apo.

For proteolytic degradation, the proteins were incubated with proteinase K (Ambion) at 65°C for 4 h. Proteinase K was then itself inactivated by incubation at 120°C for 1 h. The proteinase K/metallochaperone molar ratios used were 1:2,000 for ZnuA and 1:25 for Rv2059 for optimal degradation and inactivation. Protein degradation was verified by SDS-PAGE and revealed using Coomassie brilliant blue for ZnuA and silver nitrate for Rv2059 due to its lower molecular weight. The absence of a band of the corresponding molecular weight for each protein in the digested samples was considered proof of degradation.

The degraded proteins and their active pairs were used to reactivate PZAse-MT–Apo in independent Wayne assays as described above. A control consisting of inactivated proteinase K was added to the active metallochaperones to exclude any decrease in reactivation caused by proteinase K.

### Effect of thermally inactivated ZnuA in the reactivation of metal-depleted PZAse-MT–Apo.

ZnuA was thermally inactivated (denatured) using two methods. The first method comprised one thermal shock cycle of 100°C for 30 min followed by 1 min on ice, and the second consisted of three thermal shock cycles at 120°C for 30 min each followed by 1 min on ice. Both methods used 100 μl ZnuA (125 μM) in 1.5-ml vials. The thermally inactivated proteins were then evaluated in a Wayne reaction to assess reactivation of PZAse-MT–Apo. A control consisting of 100 μl 30 mM Tris-HCl buffer only was included.

### Structural model of ZnuA and Rv2059 and docking with PZAse-MT.

The M. tuberculosis protein Rv2059 structure was modeled with RaptorX (73) using as templates the Protein Data Bank (PDB) structures 5KZJ, 5UXS, 1PQ4, 3HJT, and 5HX7, which correspond to YfeA (Yersinia pestis), AztC (Paracoccus denitrificans), an ABC transporter (Listeria monocytogenes), Lmb (Streptococcus agalactiae), and ZnuA (*Synechocystis* sp.). The templates are metal-binding proteins that have been crystallized in complexes with Zn or Mn. We predicted the structure of the region of the protein only from amino acids 38 to 304, which corresponds to between 17 and 24% of the identity of the templates.

The PZAse and ZnuA crystal structures were obtained from the PDB (IDs 3PL1 and 2OSV) (30, 50). Protein-protein docking was performed using the HADDOCK Web server (73) to model the heterodimer structure of PZAse in complex with Rv2059. We defined the residues involved in metal binding as active residues to guide the protein-protein docking. In the case of Rv2059, the metal-binding residues were H75, H135, and E199, as predicted using the NCBI Conserved Domain Database (74). The metal-binding residues on the PZAse (PDB ID 3PL1) were D49, H51, H57, and H71 (34), and for E. coli ZnuA (PDB ID 2OSV), the residues were E77, H78, H161, and H225 (50).

### Wayne test.

The Wayne test is a colorimetric method that detects POA that is released by bacilli into the culture medium (72). The test was performed as described by P. T. Kent (75). Based on the color intensity, the selected strains were classified as having a positive, weak, or negative Wayne activity. Both the CDC1551 and CDC1551-RV2059-KO strains were tested in duplicate for each strain.

### Measurement of POA efflux by quantitative Wayne test.

The production of POA by M. tuberculosis strains CDC1551 and CDC1551-Rv2059-KO (obtained through BEI Resources) was measured using the Wayne assay in citrate buffer, pH 7.0, as described previously (76). For each strain, 3 inoculation loops of culture were resuspended in 500 μl of citrate buffer and disaggregated with 2.5-mm beads by vortex agitation. Then, 20 μl of cell suspension was added to 5 ml of citrate buffer, pH 7.0, in triplicate until McFarland scale 4 turbidity was obtained. Pyrazinamide (400 μg/ml) was then added to the suspension.

Each sample was harvested 6 times at 12-h intervals. During each harvest, cells were collected from 500 μl of sample by centrifugation at 13,000 rpm for 10 min; the supernatant was recovered, inactivated at 95°C for 20 min, and stored at −20°C. It was then incubated at 37°C with constant agitation (300 rpm). The presence of POA was revealed by adding 10% ferrous ammonium sulfate and measured at 450 nm.

### Comparative Bactec MGIT measurement.

The Bactec MGIT 960 assay is an automated assay that is used to evaluate M. tuberculosis growth and that also provides information on PZA susceptibility. The assay was performed according to standard guidelines. Briefly, a loopful of M. tuberculosis culture grown on solid 7H10 medium was suspended in a tube containing beads of 1-mm diameter. This was vortexed and incubated for 20 min, and the bacterial suspension was transferred to a separate tube, where its turbidity was adjusted to a 0.5 McFarland standard equivalent. Dilutions (1:5 and 1:50) were then prepared using saline solution, and 500 μl was added to 7H9 medium supplemented with oleic acid-albumin-dextrose-catalase (OADC), with and without 100 μg/ml PZA. The tubes were incubated in the Bactec MGIT 960 instrument. A final readout representing the ratio between the fluorescences of the strain in the presence and absence of PZA was taken to indicate whether the strain was sensitive or resistant to PZA.

### Statistical analysis.

The effects of the treatment (Zn^2+^, ZnuA, ZnuA-Apo, or Rv2059) on the activity of PZAse-EC–Apo and PZAse-MT–Apo were calculated using a multiple-linear-regression model, including an interaction term. Statistical analysis was performed under 5% significance using the statistical package Stata 13.0.
